# Rheumatoid arthritis-like active synovitis with T-cell activation in a case of idiopathic multicentric Castleman disease: A case report

**DOI:** 10.1097/MD.0000000000015237

**Published:** 2019-05-03

**Authors:** Mizuna Otsuka, Tomohiro Koga, Remi Sumiyoshi, Momoko Okamoto, Yushiro Endo, Sosuke Tsuji, Ayuko Takatani, Toshimasa Shimizu, Takashi Igawa, Shin-ya Kawashiri, Naoki Iwamoto, Kunihiro Ichinose, Mami Tamai, Hideki Nakamura, Tomoki Origuchi, Niino Daisuke, Atsushi Kawakami

**Affiliations:** a Department of Immunology and Rheumatology, Unit of Advanced Preventive Medical Sciences, Nagasaki University Graduate School of Biomedical Sciences; b Center for Bioinformatics and Molecular Medicine, Nagasaki University Graduate School of Biomedical Sciences; c Nagasaki Educational and Diagnostic Center of Pathology, Nagasaki University Hospital, Nagasaki, Japan.

**Keywords:** idiopathic multicentric Castleman disease, interleukin-6, rheumatoid arthritis, synovitis, T cells

## Abstract

**Rationale:**

Idiopathic multicentric Castleman disease (iMCD) is a systemic disease with multiple regions of lymphadenopathy and systemic symptoms and associated with rheumatoid arthritis (RA) and collagen diseases. However, few reported have described the coexistence of iMCD and RA and the mechanisms by which iMCD induces arthritis remain elusive. We experienced a rare case of iMCD, wherein the patient exhibited symptoms of polyarthritis with high-grade fever.

**Patient concerns:**

A 34-year-old woman was admitted to our hospital for further evaluation of a high fever with polyarthritis. The levels of both rheumatoid factor and anticitrullinated protein antibody were negative. ^18^F-fluorodeoxyglucose/positron emission tomography-computed tomography showed lymphadenopathy with increased fluoro-2-deoxy-d-glucose uptake. Magnetic resonance imaging and musculoskeletal ultrasonography revealed active synovitis in the hands which was consistent with RA.

**Diagnoses:**

We diagnosed iMCD based on human herpesvirus 8 negativity, HIV negativity, systemic lymphadenopathy, and pathologic findings of the lymph nodes. The patient did not satisfy the 2010 American College of Rheumatology and European League Against Rheumatism classification criteria for RA. Cytokine assay showed elevated serum levels of interleukin-17 and CXCL10, comparable to those in patients with RA.

**Interventions:**

We administered 15 mg/d of predonisolone.

**Outcomes:**

After this treatment, the patient's symptoms showed improvement. As of this writing, we tapered the prednisolone to 7.5 mg/d, and the patient's remission has been maintained for >4 months.

**Lessons:**

The present case suggests that RA-like active synovitis may coexist in iMCD, resulting from aberrant T-cell activation and histologic examination using lymph node biopsy may help enable early diagnosis of iMCD.

## 1 Introduction

Castleman disease (CD) is a rare lymphoproliferative disease; its pathology was 1st reported by Benjamin Castleman in 1956.^[1]^ CD is classified into unicentric CD (UCD) and multicentric CD (MCD), based on the number of regions of enlarged lymph nodes with characteristic histopathologic features.^[2]^ Patients with UCD typically have focal lymph nodes and are generally asymptomatic or mildly symptomatic; in contrast, MCD is a systemic disease with multiple regions of lymphadenopathy and systemic symptoms that include fever, night sweats, weight loss, and fatigue.

The etiology of MCD remains largely unclear. The observed clinical symptoms of MCD are attributed to hypercytokinemia, including elevated levels of interleukin-6 (IL-6).^[3]^ MCD can be divided into the human herpesvirus 8 infection-related MCD and idiopathic MCD (iMCD).^[4]^ Majority of the patients with UCD are treatable via surgical excision. The prognosis of iMCD varies, and the achievement of remission can be challenging.

The iMCD is associated with rheumatoid arthritis (RA) and collagen diseases^[5]^; therefore, some patients with iMCD develop arthritis. However, the mechanisms by which iMCD induces arthritis remain elusive.

We experienced the case of a patient with iMCD who developed arthralgia. This patient had active synovitis detected by magnetic resonance imaging (MRI) and musculoskeletal ultrasonography (MSUS). In this case, cytokine analysis revealed increased levels of IL-17 and CXCL10, suggesting the involvement of T-cell activation in the mechanism responsible for the development of synovitis in iMCD.

## 2 Case report

The healthy 34-year-old woman had experienced arthralgia at the shoulders, wrists, and ankles for a period of 2 months. Subsequently, she presented with a high fever and swelling of fingers and visited a local orthopedic clinic. Thereafter, she was admitted to our hospital for further evaluation of fever of unknown origin with polyarthritis in May 2018.

**Figure 1:**
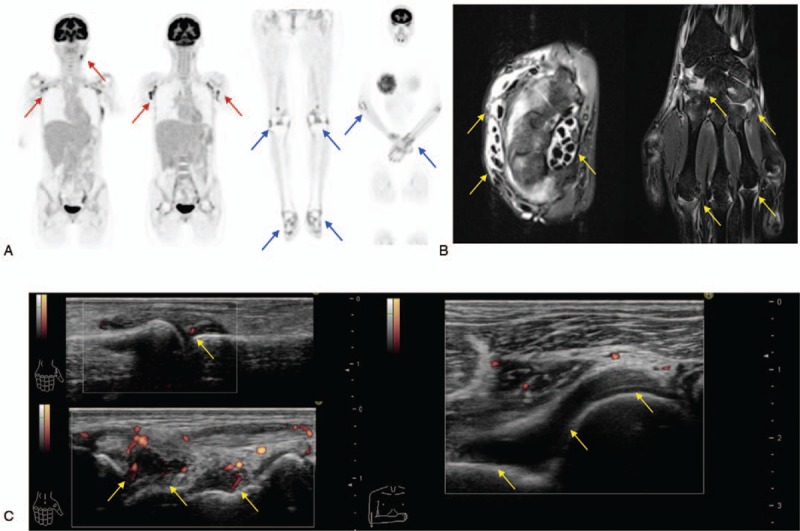
(A) Fluoro-2-deoxy-d-glucose accumulation visible in her left cervical lymph node (red arrows); axillary lymph nodes (red arrows); as well as elbow wrist, knee, and ankle joints (blue arrows). (B) Magnetic resonance imaging shows active synovitis and tenosynovitis in her right hand (yellow arrows). (C) Musculoskeletal ultrasonography assessment in the 1st metacarpophalangeal joint and carpal joints show synovial thickening with high-intensity power Doppler signals (yellow arrows). Synovial thickening is present in the humeroradial joint (yellow arrows).

**Figure 2:**
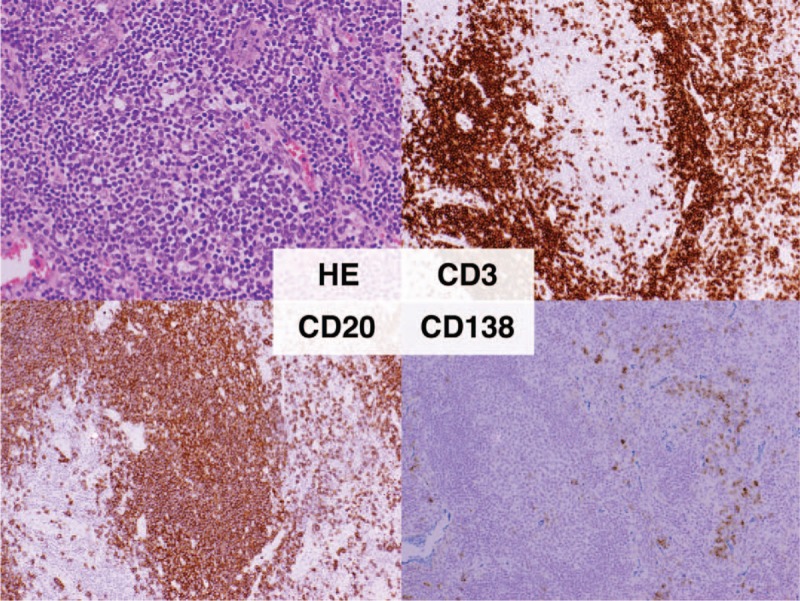
Left axillary lymph node biopsy reveals blood vessels at the atrophied germinal center with the accumulation of lymphocytes (hematoxylin and eosin [HE] staining, original magnification ×200). Immunohistochemical stains show CD3^+^ T cells, CD20^+^ B cells, and CD138^+^ plasma cells (DAB and HE staining, original magnification ×200).

On admission, her body temperature was 37.5°C, blood pressure 86/54 mm Hg, and heart rate 80 bpm. A physical examination showed swollen lymph nodes in the left neck and both axillae. Laboratory investigations (Table 1) showed reduced hemoglobin (Hb) (8.7 g/dL) and albumin levels (2.9 g/dL), with elevated serum C-reactive protein (CRP) (10.14 mg/dL), IL-6 (111.69 pg/dL), and vascular endothelial growth factor (VEGF) (202.57 pg/dL) levels. Immunologic studies showed antinuclear antibody 1280-fold (speckled type); however, all specific autoantibodies were negative. Cytomegalovirus antigenemia assay and Epstein–Barr virus (EBV) DNA were also negative. An examination with ^18^F-fluorodeoxyglucose/positron emission tomography-computed tomography (^18^F-FDG/PET-CT) showed multiple lymphadenopathy with increased fluoro-2-deoxy-d-glucose (FDG) uptake (Fig. 1A). In addition, FDG accumulation was observed in her joints (Fig. 1A). Although no erosion was detected by the X-ray in the hands and feet, MRI revealed active synovitis and tenosynovitis in the right hand (Fig. 1B). MSUS assessment of the hands, elbows, and ankles showed synovial thickening with remarkable PD signals (Fig. 1C). Left axillary lymph node biopsy revealed blood vessels at the atrophied germinal center along with the accumulation of CD3^+^ T cells, CD20^+^ B cells, and CD138^+^ plasma cells consistent with CD (Fig. 2).

**Table 1:**
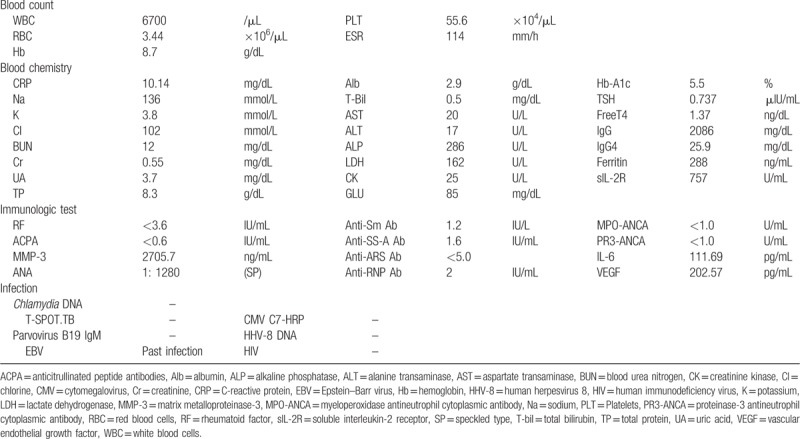
Laboratory investigations in the present case.

**Figure 3:**
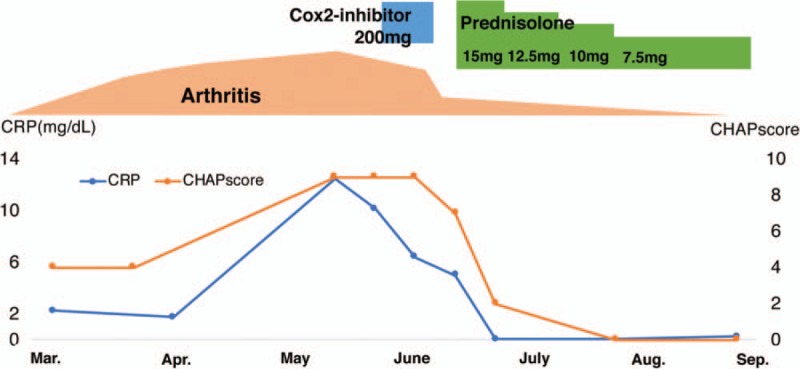
The clinical course of the present patient. The graphs display the arthritis severity, C-reactive protein (CRP) level, CHAP score, and treatment interventions.

**Figure 4:**
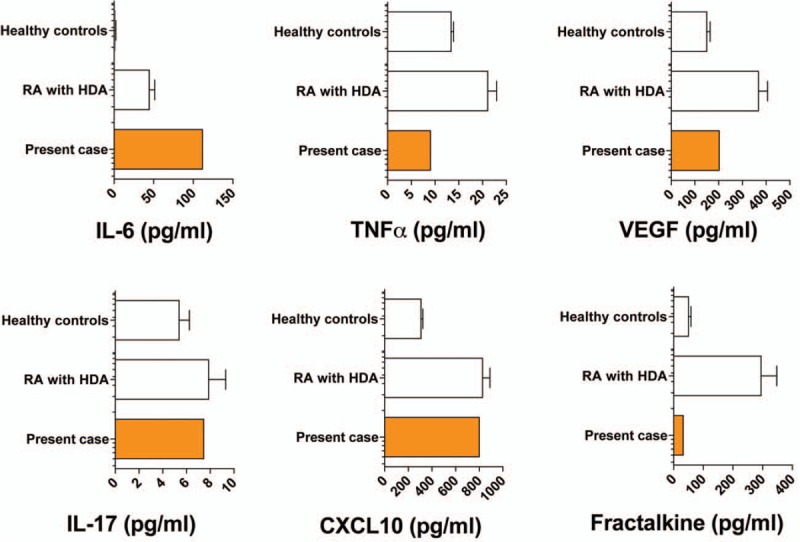
Comparison of the serum cytokine levels of patients with rheumatoid arthritis (RA) with high disease activity (HDA) and those of healthy controls (HCs). Mean ± standard error of mean (SEM); RA with HDA: n = 83; HCs: n = 133. IL = interleukin; TNFα = tumor necrosis factor alpha; VEGF = vascular endothelial growth factor.

We made a diagnosis of iMCD on the basis of international, evidence-based consensus diagnostic criteria.^[6]^ We excluded infection, malignancy, and autoimmune disorders that can mimic iMCD. The disease severity in this patient was classified as mild based on the criteria proposed in Japan.^[7]^ The patient's findings did not fulfill the 2010 American College of Rheumatology and European League Against Rheumatism classification criteria for RA.^[8]^ Accordingly, we initiated 15 mg/day of prednisolone (PSL) in mid-June 2018, and it reduced the arthritis, fatigue, fever, and lymphadenopathy as well as normalized the levels of Hb, albumin, and CRP. At the time of writing this report, we tapered the PSL to 7.5 mg/day, and the patient's remission has been maintained for >4 months. The clinical course of the patient has been shown in Figure 3. We evaluated her disease activity based on the recently proposed CHAP score.^[7]^ We investigated her serum cytokine levels at diagnosis before PSL initiation. Her IL-6, IL-17, and CXCL10 levels were higher than those of healthy controls; however, the tumor necrosis factor alpha, VEGF, and fractalkine levels did not show such a trend (Fig. 4).

## 3 Discussion

We experienced a rare case of iMCD, wherein the patient exhibited symptoms of polyarthritis with high-grade fever and was treated successfully with a low dose of PSL. She presented with RA-like active synovitis in her hands. To our knowledge, none of the reviews on rheumatic diseases complicated with CD have reported the case of a patient with iMCD complicated with RA.^[9]^ Further, to the best of our knowledge, no investigations have been performed to evaluate arthritis in iMCD patients using FDG/PET-CT, MRI, and MSUS. Synovitis detected using MRI or MSUS is useful for the early diagnosis of RA.^[10]^ It is noteworthy that based on some reports, PD signal positive synovitis predicts future RA development in patients with undifferentiated arthritis.^[11,12]^ MSUS and MRI findings of the synovitis in her fingers and wrist joints were consistent with RA. Therefore, although her synovitis disappeared rapidly following treatment with PSL alone without subsequent relapse, she may develop RA in the future.

We compared the serum cytokine levels of this patient and patients with RA. The serum IL-6 level was higher in our patient than those in patients with RA with high disease activity; moreover, the concentrations of IL-17 and CXCL10 were comparable to those of patients with RA. CXCL10 (also known as interferon-γ-inducible 10-kD protein [IP-10]) is a chemokine that may play a role in the immunopathogenesis of RA.^[13]^ CXCR3, preferentially expressed on Th1 cells, is the receptor of CXCL10, suggesting that CXCL10 induces the migration of Th1 cells.^[13,14]^ Therefore, we believed that the Th1 cells as well as the activation of Th17 cells contributed to active synovitis in the present case.

The IL-6 is an inflammatory cytokine that is implicated in the pathogenesis of both, iMCD and RA^[15]^; however, active arthritis is rare in patients with iMCD. In fact, IL-6 transgenic mice do not exhibit symptoms of active synovitis with bone erosion.^[16]^ In this case, we observed elevated cytokines related to T-cell activation, indicating that T cells may have contributed to the development synovitis in combination with IL-6. Consistent with the finding of a large number of CD3^+^ T cells in the lymph node in our patient, T cells are reportedly activated in the lymph nodes in patients with iMCD.^[17]^ Thus, the role of T cells in the development of arthritis should be investigated in the future.

To conclude, we experienced the case of a patient with iMCD that developed with RA-like active synovitis and was detected using MRI and MSUS. Active arthritis is rarely observed in iMCD; therefore, the activation of T cells with increased IL-6 levels was considered to contribute to the development of arthritis in this case. The present case suggests that RA-like active synovitis may coexist in iMCD and histologic examination using lymph node biopsy may help enable early diagnosis of iMCD. Further case studies and functional studies are warranted to clarify the mechanisms underlying the development of active arthritis in patients with iMCD.

## Acknowledgment

The authors thank Kaori Furukawa (Research Assistant, Department of Immunology and Rheumatology, Division of Advanced Preventive Medical Sciences, Nagasaki University Graduate School of Biomedical Sciences, Nagasaki) for her technical assistance.

## Author contributions

**Investigation:** Mizuna Otsuka, Remi Sumiyoshi, Momoko Okamoto, Yushiro Endo, Sosuke Tsuji, Ayuko Takatani, Toshimasa Shimizu, Takashi Igawa, Shin-ya Kawashiri, Naoki Iwamoto, Kunihiro Ichinose, Mami Tamai, Hideki Nakamura, Tomoki Origuchi, Niino Daisuke, Atsushi Kawakami.

**Writing – original draft:** Mizuna Otsuka, Tomohiro Koga.

**Writing – review & editing:** Tomohiro Koga.
